# Exploring the Potential of Oral Microbiome Biomarkers for Colorectal Cancer Diagnosis and Prognosis: A Systematic Review

**DOI:** 10.3390/microorganisms11061586

**Published:** 2023-06-15

**Authors:** Roxana Loriana Negrut, Adrian Cote, Adrian Marius Maghiar

**Affiliations:** 1 Department Medicine, Doctoral School of Biomedical Sciences, Faculty of Medicine and Pharmacy, University of Oradea, 410087 Oradea, Romania; popa_roxana_l@yahoo.com (R.L.N.); amaghiar@gmail.com (A.M.M.); 2 County Clinical Emergency Hospital Bihor, 410087 Oradea, Romania; 3 Department of Surgical Disciplines, Faculty of Medicine and Pharmacy, University of Oradea, 410073 Oradea, Romania

**Keywords:** colorectal cancer, oral microbiota, biomarker, periodontal pathogens

## Abstract

There is growing evidence indicating that the oral microbiota, specifically certain periodontopathogens such as *Fusobacterium nucleatum*, may play a role in the development of colorectal cancer and that it could potentially be used as a biomarker for diagnosing colorectal cancer (CRC). The question beneath this systematic review is whether the development or progression of colorectal cancer can be attributed to the presence of certain oral bacteria, which could be used for discovering non-invasive biomarkers for CRC. This review aims to give an overview of the actual status of published studies regarding the oral pathogens related to colorectal cancer and assess the effectiveness of the oral microbiome derived biomarkers. A systematic literature search was performed using four databases, Web of Science, Scopus, PubMed, and Science Direct, on the 3rd and 4th of March 2023. The studies that did not have matching inclusion/exclusion criteria were winnowed out. A total of fourteen studies were included. The risk of bias was performed by using QUADAS-2. After assessing the studies, the general conclusion is that oral microbiota-based biomarkers can become a promising non-invasive tool for detecting CRC, but further research is needed in order to determine the mechanisms of oral dysbiosis in colorectal carcinogenesis.

## 1. Introduction

Colorectal cancer (CRC) represents a public health concern, its incidence being on the 3rd place worldwide in men and 2nd in women, according to World Cancer Research Fund International, representing 10.7% of all cancers. In 2020, in Romania, the highest cancer rate for men and women combined was at 255.9 people per 100,000. Regarding the overall mortality rate from CRC in 2020, Romania is in the 9th place on a global scale [1]. A few studies have investigated the association between oral pathogens and the onset of colorectal cancer.

When CRC is detected in early stages, the five-year survival rate is over 80%, while, during advanced stages, this rate drops below 10% [2]. The fecal immunochemical test (FIT), used as a non-invasive screening method, is implemented to find high-risk individuals. However, the sensitivity of FIT is low, and it could misplace one-third of CRC from early stages [3]. The guaiac fecal occult blood test (gFOBT) is a fast, non-invasive test that has limited sensitivity for advanced colorectal neoplasms [4]. The FIT-DNA test even has a higher rate of false positive results [5,6]. For these reasons, more reliable biomarkers are required in addition to the existing ones.

The largest and most complex group of bacteria that inhabit the human body are found within the human gastrointestinal tract [7]. Generally, these bacteria are considered commensal or symbiotic. There is increasing evidence to support the hypothesis that the human gut microbiome may have a significant impact on the role in the development and progression of cancer, as well as the effectiveness of cancer treatments [8,9,10,11,12,13,14].

The quantity of saliva produced per day by an adult is on average over 1000 mL of saliva and almost all of it enters the gastrointestinal tract, this being inoculated daily by approximately 1011 bacteria from the oral cavity, many of them proven to be detected also in the fecal microbiota of approximately 45% tested individuals [15,16].

Heightening evidence shows that the oral microbiome is capable of ectopic colonization and can produce a wide range of microbial metabolites that have the potential to promote carcinogenesis through modulation of pathways related to energy homeostasis, food intake, and immunologic balance [17,18,19]. The gut microbial composition and function can be influenced by numerous environmental factors, including diet and lifestyle. These alterations may affect host gene expression, metabolic regulation, and local and systemic immune responses, potentially impacting cancer development. [20] Microbes are known to act as chemical converters, metabolizing nutrients acquired from the diet or the ones produced by the host organism [21]. The metabolites produced by the bacterial activity can influence the development of tumors and promote genotoxicity or tumor suppression through different mechanisms [22]. Consequently, oral microbial communities can have an impact on the structure of the gut microbial community [23]. The term “microbiota dysbiosis” refers to changes in bacterial composition [24], and investigating both oral and intestinal microbiota is highly significant for understanding the mechanisms behind colorectal cancer development [8,25,26,27].

The latest hypothesis suggests that the oral microbiome may serve as a potential biomarker for detecting CRC, supported by a few studies demonstrating the presence of oral bacteria in saliva, feces, and even intra-tumoral samples. The scope of this systematic review is to provide a thorough analysis of the status of biomarkers derived from the oral microbiome.

## 2. Materials and Methods

### 2.1. Search Strategy

A search of the existing literature was conducted in March 2023, using the databases Web of Science, PubMed, Scopus, and Science Direct. The following terms were used: colorectal cancer, colonic cancer, oral microbiota, oral microbiome, and periodontal pathogens. The search was carried out by using Boolean operators (AND, OR), round and square brackets to group search terms and determine the order of operations in a search query, and field-specific operators such as title, abstract, and title-abstract. Only original articles were included in the search, with any other type of publication being excluded by using the website filters.

The literature review was conducted in accordance with the Preferred Reporting Items for Systematic Reviews and Meta-Analyses (PRISMA) guidelines [28]. The entire search strategy can be found in Supplementary File S1. The protocol was registered in PROSPERO database CRD42023410069.

### 2.2. Study Selection

The records underwent an independent screening process by the two authors (N.R. and C.A.) based on the title and abstract and assessed for inclusion based on the full text. For the evaluation of the records, the Rayyan platform (Quatar Computing Research Institute) was used to perform a blind evaluation based on the title, abstract, and key-words made by the authors. Disagreement of the records screened was solved by discussion or by consulting a third reviewer (M.A.). The difference of opinion regarding the full text screening was solved in the same manner. 

### 2.3. Eligibility Criteria

The articles were included for assessment if they evaluated whether the oral microbiome is distinctive and predictive in CRC or appraise the oral microbiome derived biomarkers for CRC detection, including any stage 0/I/II/III/IV. The studies that assess oral microbiota in CRC by comparing with precancerous lesions patients as well as with a health control group were included and only the interest information was extracted.

Articles were excluded on the basis of certain criteria:

1. Viral, fungal, and immunological biomarkers;
2. Disease other than CRC;
3. Inflammatory bowel disease (IBD)-related CRC or CRC as part of a syndrome;
4. In vitro or animal studies;
5. No full text available;
6. Case report or case series;
7. Questionnaire based study; computer simulations;
8. Review or meta-analysis;
9. Drug impact on the gastrointestinal microbiome;
10. No data of interest for the current review;
11. Only precancerous lesions: complex adenoma, high-grade dysplasia, and carcinoma in situ (CIS);
12. Immunological pathways to cancer progression;
13. Biomarkers assessed after surgical treatment.

### 2.4. Data Extraction 

Data extraction from the eligible articles was performed by the two authors (N.R. and C.A.) independently and was confirmed by the third author (M.A.). The extracted data included: author, year of publication, country, number of participants (total and in each group), comparison groups, stage of disease, number of participants by each CRC stage where mentioned, sample origin, biomarker found, technique used for discovery, and performance of the discovered biomarker based on AUC (area under curve) and *p*- value. 

### 2.5. Comparison with Other Groups

Comparison with a group of healthy controls was based on colonoscopy or defined group without prior gastro-intestinal (GI) diseases. Comparison with the non-cancerous lesion defined by colonoscopy and histopathologic examination (Adenoma or Hyperplastic Polyp).

### 2.6. Assessment

Each study was independently assessed for the risk of bias and applicability by the authors, by using the QUADAS-2 (Quality Assessment of Diagnostic Accuracy Studies checklist), by evaluating the four domains regarding the risk of bias (patient selection, index test, reference standard, flow, and timing), and by three domains related to concerns regarding applicability (patient selection, index test, and reference standard) [29]. The QUADAS-2 was assessed in RevMan 5.4 [30].

## 3. Results

### 3.1. Study Selection

**Figure 1:**
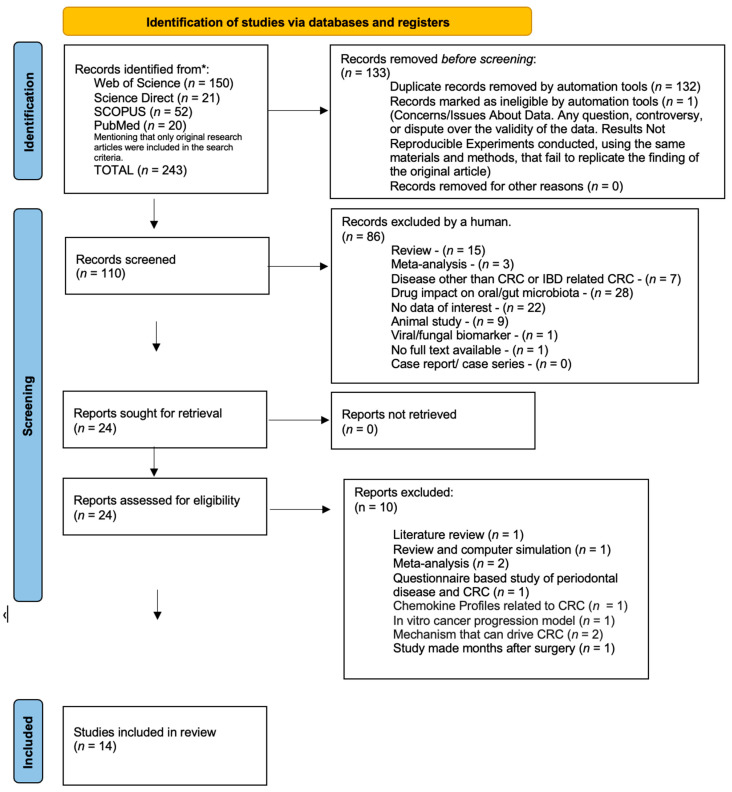
PRISMA flow diagram of studies selection. * Consider, if feasible to do so, reporting the number of records identified from each database or register searched (rather than the total number across all databases/registers).

A systematic literature search was made using four databases: Web of Science, PubMed, Scopus, and Science Direct. As mentioned before, the search was carried out by using Boolean operators (AND, OR), round and square brackets to group search terms and determine the order of operations in a search query, and field-specific operators such as title, abstract, and title-abstract. We mention that only original articles were included, with any other type of publication (review article, early access, meeting abstract, proceeding paper, etc.) being excluded by using website filters. A total of 243 records were identified, 150 from Web of science, 21 from Science Direct, 52 from Scopus, and 20 from PubMed. Several duplicates (nr. 132) were removed by automation tools, and one was marked ineligible. After duplicates were eliminated using automated tools, 110 records were screened by title and abstract, resulting in another 86 excluded records. A total of 24 studies were assessed for eligibility based on full-text screening. Following a thorough study selection process, a total of 14 studies were selected for this systematic review. The PRISMA guidelines were followed during the selection process [28] and are depicted in Figure 1—PRISMA flow diagram.

### 3.2. Study Characteristics

Two studies were conducted in the Middle East; three of the studies were conducted in the United States and Canada; three were conducted in Europe, and six were conducted in Asia. The number of total inclusions in the studies varied from 692 participants to 20, for CRC from 511 to 10, for adenoma from 21 to 43, and for healthy control group from 10 to 461. Some of the studies compared colorectal cancer with a heathy control group [31,32,33,34,35,36]; others compared it to a healthy control as well as with benign lesions group [33,37,38] or only to adenoma [39], while other studies researched only colorectal cancer and made no comparison with a control group [40,41]. One of the studies [34] also evaluated a comparison between early and advanced stages. The samples used in the included studies were saliva, stool, and colonic or tumor mucosa. Even though we are investigating a non-invasive biomarker, we considered the studies that involved the colonic mucosa/tumor as well for the grounds of evaluating how the oral bacteria contaminate the tumoral tissue compared to stool or saliva. Main characteristics of the studies are detailed in Table 1.

Where data were available, the median age and percentage of male for each group was extracted. Some studies reported only the age category of the participants and not the median age [32,35,38,43]. Only two articles did not mention anything regarding the stages of CRC included in the study [42,43]. Seven studies mentioned the number of cases included in each stage [34,36,37,38,39,40,41], while other three mentioned the stages included in the study but did not mention the number of participants included from each stage [32,33,44]. All the studies included participants diagnosed in early stages, as well as advanced stages, except two articles: one study by Mark Loftus et al. [31] that researched only advanced stages III and IV and another by Rezasoltani Sama et al. [32] that included only early stages 0 and I. See Table 2.

The comparation of interest is represented by CRC cases and healthy controls. The article written by Xin Zhang et al. [38] compared the CRC cases with a control group represented by healthy participants, as well as patients diagnosed with adenoma or hyperplastic polyp that were all included in the control group. Two articles had no comparation group and researched the oral bacteria from the CRC group [40,41]. Hoang, N.H. et al. [39] evaluated CRC group in comparation with adenoma group. Katushiko et al. [40] was the only study that set side by side the early stages and advanced ones and evaluated the oral microbiota.

The main analytical method used was DNA extraction and 16S rRNA sequence [32,33,34,37,42,43]. Two studies used WGS [31,39], and one used NGS [35]; five used qPCR [35,36,40,41,44]; one [38] used multiplex qPCR. The diagnostic performance was evaluated by AUC (area under curve—95% CI), that was mentioned only in five studies [31,33,36,37,38] and showed an AUC ranging from 0.83 to 0.94, suggesting a high accuracy for the use of oral microbiome derived biomarkers. The *p*-value for the potential biomarkers used is significant in most of the studies. Table 3 presents the details of each study.

The WGS (whole-genome shotgun sequence) was used in only two studies; one of them used fecal samples collected from CRC group and healthy controls [31] while the other [39] used saliva and gut tissue samples from the participants diagnosed with CRC or polyps.

In the study conducted by Mark Loftus et al. [31], they examined fecal samples from healthy individuals and those with late-stage colorectal cancer (CRC) and revealed important insights into the role of the gut microbiome in CRC. The results showed that the bacterial community diversity was significantly greater in the CRC group compared to the healthy group, with significantly elevated levels of 10 species of oral microbes within the CRC-associated gut microbiome. These findings suggest that oral microbes may play a crucial role in the development and progression of CRC. One of the most significant findings of the study was the identification of the 10 species of oral microbes that were significantly elevated in relative abundance within the CRC-associated gut microbiome. The 10 species significantly elevated in relative abundance within CRC were *Parvimonas micra* (qvalue = 3.09 × 10^−9^), *Peptostreptococcus stomatis* (qvalue = 4.51 × 10^−8^), *Gemella morbillorum* (qvalue = 4.55 × 10^−8^), *Fusobacterium nucleatum* (qvalue = 1.08 × 10^−6^), *Streptococcus anginosus* (qvalue = 1.13 × 10^−3^), *Dialister pneumosintes* (qvalue = 1.37 × 10^−3^), *Peptostreptococcus anaerobius* (qvalue = 4.74 × 10^−3^), *Streptococcus* sp. KCOM 2412 (*Streptococcus periodonticum*) (qvalue = 7.18 × 10^−3^), *Ruminococcus torques* (qvalue = 1.55 × 10^−2^), and *Filifactor alocis* (qvalue = 2.85 × 10^−2^). The eight Highly prevalent species unique to the CRC associated group were *Intestinibacillus massiliensis*, *Prevotella copri*, *Haemophilus parainfluenzae*, *Ruminococcus bicirculans*, *Streptococcus mitis*, *Neglecta timonensis*, *Bifidobacterium catenulatum*, and *Anaerotignum neopropionicum*. It is important to mention that *Streptococcus mitis* and *Haemophilus parain-fluenzae* are both classified by the eHOMD as oral microbes. The majority (approximately 82% on average) of a sample’s total relative abundance was attributed to HPS [31].

The study of Hoang N. Tran et al. [39] found that tumor-enriched taxa include bacteria of oral origin, such *Gemella, Peptostreptococcus*, *F. nucleatum*, *Leptotrichia*, *Selenomonas sputigena*, and *Campylobacter rectus*. These bacteria were overabundant in the tumor microbiomes, compared to control group, with a *p*-value < 0.05. The results of the study also show that *F. nucleatum* was enriched in advanced CRC stages with significant *p*-value < 0.05. The study applied anaerobic culturing and whole genome sequencing (WGS) to isolate and identify Fusobacterium subspecies from gut tissues, which uncovered novel subspecies of both *F. nucleatum* and *F. periodonticum*. However, the approach generally has low sensitivity, and bacterial recovery is subjected to factors such as storage time and condition, which cannot capture the high diversity of *Fusobacterium* in the oral niche [39].

In the study of multiplex qPCR by Xin Z. [38], the authors evaluated the potential use of salivary *Fn* DNA as a biomarker for colorectal cancer (CRC). First, they evaluated the stability of candidate reference genes in saliva using NormFinder and geNorm algorithms and identified GAPDH and TERT as the most stable reference gene and developed a multiplex qPCR method to detect salivary *Fn* DNA and found that the level of salivary *Fn* DNA was significantly elevated in CRC patients compared to healthy controls, hyperplastic polyps (HP), and patients with adenomas (Ad). Moreover, the level of salivary *Fn* DNA was positively correlated with the TNM stage of CRC. ROC analysis showed that salivary *Fn* DNA had an AUC of 0.841 for the diagnosis of CRC, with a sensitivity of 71.5% and a specificity of 82.1%. The diagnostic performance of salivary *Fn* DNA was superior to that of traditional serum tumor markers [38].

Zhang, S. et al. [37] aimed to investigate the differences in the oral microbiome of individuals with colorectal adenoma (CRA), colorectal cancer (CRC), and healthy controls and to identify potential biomarkers for the early detection of CRA and CRC by collecting oral swab samples. The samples underwent DNA extraction and 16S ribosomal RNA gene sequencing. The abundance of certain bacteria, including Fusobacteria and Bacteroidetes phyla, and the genera *Fusobacterium*, *Prevotella*, and *Veillonella*, were higher in the CRC group than in the healthy control group. Additionally, two types of oral bacterial co-abundance groups (CAGs) were identified: the pathogen CAG and the biofilm CAG. A random forest classifier model was constructed to distinguish the CRC group from the healthy control group, and five out markers were identified as the optimal marker set: *Cyanobacteria*, *Veillonella*, *Fusobacterium*, *Selenomonas*, and *Gemella*. The POD index achieved an AUC value of 76.42% in the discovery phase and an AUC value of 83.74% in the validation phase, indicating the potential of oral microbiota-based biomarkers for the non-invasive detection of CRA and CRC [37].

The study of Rezasoltani Sama [32] investigated the differences in microbial community composition and diversity between colorectal cancer (CRC) patients and healthy controls (HCs) using saliva and fecal specimens. The relative abundance of bacterial genera was analyzed, and it was found that CRC patients’ fecal specimens had the highest abundance of the genera *Ruminococcus-torques-group*, *Granulicatella*, and *Ruminococcus-gauvreauii-group*. HC saliva samples were found to be more biodiverse than CRC saliva samples, while CRC fecal samples appeared to be more enriched than their HC counterparts. The study also looked at phenotype-microbial associations and used a random forest model for biomarker discovery to differentiate between phenotypes. The CRC group saliva was characterized by the top three genera that were differentially abundant—*Eubacterium* spp., *Bifidobacterium* spp., and *Fusobacterium* spp [32].

The research made by Burkhardt Flemer [33] analyzed the microbiota from individuals with CRC, colorectal polyps, and healthy controls from multiple body sites using 16S rRNA gene amplicon sequencing. The researchers found that the overall oral profile of bacterial OTUs was significantly different between individuals with CRC and healthy controls. The researchers found the 10 of the most abundant bacterial genera across oral swab samples: *Streptococcus* (30.7% of all assigned reads), *Haemophilus* (14.2%), *Neisseria* (8.8%), *Prevotella* (6.6%), *Fusobacterium* (5.4%), *Veillonella* (5.4%), *Leptotrichia* (3.9%), *Rothia* (3.9%), *Actinomyces* (2.9%), and *Porphyromonas* (2.4%). A number of 17 OTUs were shared between the oral cavity and CRC and polyp samples. The study confirmed the predictive value of the oral microbiota for CRC screening and found that oral bacteria are abundant in the gut microbiota”of i’dividuals with CRCs and polyps and form similar co-abundance networks on both oral mucosa and colonic tissue [33].

The study by Yao Wang [42] aimed to investigate the differences in the microbiota composition and diversity of colorectal cancer (CRC) patients and healthy controls (HC) also by using 16S rRNA gene sequencing. They included a total of 30 CRC patients and 30 HC patients in the study and collected matched unstimulated saliva, mucosal biopsy or carcinoma tissue, and stool samples. The microbial diversity of CRC patients was compared to that of HC patients based on Simpson, Sobs, Ace, Shannon, and Chao indices. The results showed that the salivary microbiome of CRC patients had statistically higher alpha-diversity than HC, while the mucosal microbiome in CRC patients had statistically lower richness and diversity compared to that in the HC group. The beta-diversity for the salivary and mucosal microbiome was also clearly separated between the two groups, while there was no significant difference in the stool microbiome. They also compared the relative abundance of the 10 most abundant microbial taxa in each group and found that the salivary level of *Porphyromonas gingivalis* was significantly elevated in CRC patients (Mann–Whitney U test *p* value = 0.013). Firmicutes and Bacteroides in the mucosa microbiome were more abundant in CRC, although the difference in Firmicutes did not reach statistical significance. The researchers clustered the oral and mucosa microbiome into different oral types and enterotypes. They found that CRC was dominated by type II oral-type: *Streptococcus*, *Neisseria*, *Porphyromonas*, *Prevotella_7*, and *Haemophilus genus* and by type III enterotype, which was represented by *Fusobacterium*, *Bacteroides*, *Streptococcus*, and *Peptostreptococcus* [42].

In the study made by Yaohua Yang [43], they aimed to investigate the associations of overall microbiome composition and some oral bacterial taxa with colorectal cancer (CRC) risk. The overall microbiome composition was found to be similar between CRC cases and controls. However, five oral pathogens were found to be more prevalent among CRC patients than controls, with *Treponema denticola* and *Prevotella intermedia* being significantly associated with increased CRC risk with a *p* value under 0.05. Several bacterial taxa from different phyla were also associated with CRC risk. The phylum Actinobacteria family *Bifidobacteriaceae* was associated with an increased risk of CRC with a *p* value of 0.03; also, the phylum *Bacteroidetes* species *Prevotella melaninogenica* was associated with a *p* value of 0.04. Within this phylum, another two species, *Prevotella denticola* and *Prevotella* sp. oral taxon 300, were associated with an increased risk, with *p* values of 0.02 and 0.04, respectively. In the phylum *Firmicutes*, seven taxa were found to be associated with CRC risk. Among them, the most abundant taxon at the species level, Streptococcus sp. oral taxon 058, showed the most significant *p* value of 7.87 × 10^−3^. After Bonferroni correction, no taxa maintained a significant association with CRC risk [43].

Yoshinori Uchino et al. [34] compared the oral and intestinal microbiota of healthy individuals and those with colorectal cancer (CRC) to investigate the role of the microbiota in CRC. The study analyzed the composition of the microbiota of each group of samples and performed weighted UniFrac distance analysis to reveal the difference between the two groups. The results showed that the microbiota compositions in the saliva and stool samples were different. The study also used LEfSe analysis to identify microorganisms that were more abundant in patients with CRC compared to controls. The results showed that *P. stomatis*, *S. anginosus*, *S. moorei*, and *S. koreensis* were more abundant in the saliva and stool of patients with CRC compared to those in controls with statistical significance. Overall, the study suggests that certain microorganisms may contribute to the development of CRC and that maintaining good oral hygiene may reduce the risk of CRC [34].

Amal Idrissi Janati [44] analyzed biospecimens (saliva and colorectal mucosa) from all participants using qPCR to detect and quantify levels of *F. nucleatum*. The study found that *F. nucleatum* was detected in almost all saliva samples, with higher levels in both cases than controls. The study also found a low *Fn* detection in colorectal mucosa specimens in the controls and even lower in the cases [44].

Katsuhiko Nosho [40] found an association between highly enriched *F. nucleatum* in colorectal carcinoma tissues. The *F. nucleatum* positivity was detected in 8.6% of the Japanese patients with colorectal cancer. The study highlights the potential for targeting microbiota, immune cells, and tumor molecular alterations for colorectal cancer prevention and treatment [40].

Edda Russo et al. [35] aimed to analyze the bacterial communities in the oral cavity and gut of patients with colorectal cancer (CRC) compared to healthy controls. The study used high-quality 16S rRNA sequencing data from fecal and saliva samples of 10 CRC patients and 10 healthy controls. The dominant bacterial phyla were *Firmicutes* (39.18%), *Bacteroidetes* (30.36%), and *Proteobacteria* (10.65%). *Proteobacteria* and *Fusobacteria* were more abundant in cancer specimens, while *Firmicutes* and *Fusobacteria* were observed with higher abundance in the stools of CRC patients. The saliva samples were enriched in *Actinobacteria*, *Saccharibacteria*, *Proteobacteria*, *Fusobacteria*, *Firmicutes*, and *Bacteroidetes* [35].

The study of Pamela Pignatelli [41] investigated the presence of two types of bacteria, *Fusobacterium nucleatum (Fn)* and *Porphyromonas gingivalis (Pg)*, in the oral cavity, colon cancer tissue, and adjacent non-neoplastic mucosa of 36 colon cancer patients using quantitative real-time PCR (qPCR). The study found that *Fn* was present in both the oral cavity and matched cancer tissue and adjacent non-neoplastic mucosa, while Pg was present only in the oral cavity and absent in a representative series of colon tissues. There was a significant increase in the amount of *Fn* present in the colon cancer tissue during the advanced stages of colorectal cancer in such a way that this increase was predictive of the staging with a *p*-value of 0.016. The study found a moderate positive correlation (*p* = 0.056) between the *Fn* quantity in oral tissue and tumor tissue [41].

Deniz Can Guven et al. [36] analyzed saliva samples from 148 cases, 71 patients diagnosed with CRC and 77 healthy controls. The study used qPCR to examine the prevalence and amount of *Fusobacterium nucleatum (Fn)*, *Porphyromonas gingivalis (Pg)*, and *Streptococcus gallolyticus (Sg).* Saliva samples of CRC patients had higher amounts of *Fn* (*p* = 0.001) and Sg (*p* < 0.001) compared with the healthy control group. *Fn* was detected in 97.2% cases in the CRC group and 96.1% in the control group (*p* > 0.99). The mean *Fn* was higher in-patient saliva samples compared with controls (*p* = 0.001). Sg was detected in 31% of patient CRC group and in 27.3% of the control group. *Pg* detection rates and amount were similar in both groups (*p* = 0.917) [36].

### 3.3. Risk of Bias and Applicability

**Figure 2:**
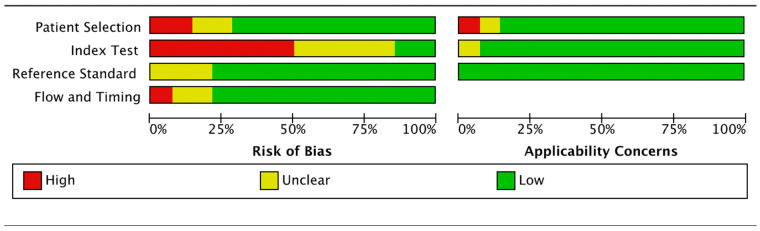
Risk of Bias and Applicability.

To assess the risk of bias and applicability, the QUADAS-2 tool from RevMan 5.4 was used. For the first domain, “patient selection”, the overall risk of bias was high in two studies, unclear in two, and low in 10. For the second domain “index test”, the risk of bias was assessed as high in seven studies, low in two, and unclear in five. The “reference test” showed a low risk of bias with three unclear, zero high-risk, and eleven low-risk. The domain “flow and timing” was identified as a low risk in eleven studies, high risk in one, and of unclear risk in two. The applicability concern was low, showing a high degree of applicability since the articles were preselected. The results are presented in Figure 2 and also in Supplementary File S2.

## 4. Discussion

Fourteen publications examining the correlation between oral microbiome and colorectal cancer were reviewed. Due to variations in cohort sizes, demographics, analytical methods, and statistical models, a meta-analysis was not possible, as the data and interpretation of results could not be standardized. As a result, the findings were presented solely in a descriptive form.

While these articles demonstrate the potential of oral microbiome-derived biomarkers in the diagnosis of colorectal cancer, more research is needed to further validate their diagnostic accuracy, as well as to standardize the methods of collection and analysis of oral microbiome data.

The publications collectively suggest that certain bacterial species, particularly those of oral origin, may be associated with CRC development and can potentially serve as diagnostic biomarkers for early CRC detection.

The diagnostic performance showed a confidence interval between 0.83 and 0.94 but was assessed only in five of the studies. The studies suggest that oral bacteria derived biomarkers could be used to augment the current screening tests.

A few of these papers were neither designed to achieve a primary diagnostic endpoint nor conducted in populations that are clinically representative. Several studies did not differentiate between different stages or anatomical locations, while some of them even combined different stages to generate a general diagnostic summary.

An AUC of 0.94 was found to be the optimal diagnostic performance across the studies and analyzed the microbiota from individuals with CRC, colorectal polyps, and healthy controls from oral swab, stool, and colonic mucosa sites using 16S rRNA gene amplicon sequencing.

The majority of this research papers have focused on bacterial taxonomy as it is currently the most standardized technique in this field, primarily relying on the 16S rRNA gene. Investigating a more accurate and sensitive biomarker for the prediction of CRC requires exploring the functional level, which can provide insight into microbiome changes associated with the development of CRC. Thus, identifying functional biomarkers may be a more effective approach for the detection of CRC [45].

The gut microbiome Is highly variable and heterogeneous between individuals, influenced by various factors such as age, gender, BMI, diet [46,47], and antibiotic use [48,49]. When comparing healthy controls and colorectal patients, it is important to incorporate these factors into the analysis by matching the groups. However, most papers included in the analysis did not implement this and only reported potential influencing factors on the microbiome composition.

The diversity of the oral–fecal microbiome varies between geographically discrete populations and across countries; most of the included studies were performed in Asia, which limits their translation to other regions. Another major limitation of these studies is the lack of standardization in sample collection and processing. Sample collection also varied widely between studies. DNA extraction was carried out using a range of extraction kits, adding an additional variable that influenced the outcome of the microbiome composition. The analytical approaches employed by the included articles also lacked standardization. The researchers used different analytical methods to identify bacteria, including 16S rRNA, qPCR, or WGS. Within these methods, there was again a large heterogeneity in the choice of primers used, leading to potential biases and making comparisons between them almost impossible.

## 5. Conclusions

The studies included in the actual review provide evidence of the potential role of oral microbiota in the development and progression of CRC. The identification of microbial biomarkers, particularly salivary *Fn* DNA, may offer new avenues for the development of non-invasive screening and diagnostic tools for CRC. In line with previous findings, it has been observed that oral periodontopathic bacteria have the capacity to migrate to the colon, disrupting the microbiota and resulting in dysbiosis, compromising the colonic integrity barrier, and leading to heightened levels of toxic metabolites and proteolytic activity, factors that contribute to inflammation and the development of colorectal cancer [50].

Different studies identified various bacterial taxa associated with CRC, including *Fusobacteria*, *Bacteroidetes*, *Prevotella*, *Veillonella*, and *Treponema denticola*. The abundance and composition of oral microbiota were found to be different between CRC patients and healthy controls. These findings suggest that the oral microbiome, particularly certain bacterial species, may play a crucial role in the development, progression, and potential diagnosis of CRC.

In the current research study, a meta-analysis could not be conducted due to significant variations in study design, conceptual differences, and analytical precision. Therefore, we consider a systematic review approach to be more suitable.

Further CRC microbiome large-scale validation studies are needed to confirm these findings before being able to translate them into clinical practice.

## Figures and Tables

**Table 1:** Study characteristics.

Author	Ref. Nr.	Year	Country	Sample	Participants	Nr. CRC/CRA/HC
Zhang S. et al.	[37]	2020	China	S	253	161/34/58
Loftus M. et al.	[31]	2021	USA	F	252	74/N/178
Rezasoltani S. et al.	[32]	2022	Iran	S/F	40	Not Specified
Flemer B. et al.	[33]	2017	Ireland	S/CM/F	234	99/32/103
Wang Y. et al.	[42]	2021	China	S/CM/F	60	30/N/30
Yang Y. et al.	[43]	2019	USA	S	692	231/N/461
Idrissi J. et al.	[44]	2022	Canada	S/CM	43	22/N/21
Nosho K. et al.	[40]	2016	Japan	CM	511	511/0/0
Uchino Y. et al.	[34]	2021	Japan	S/F	103	52/N/51
Russo E. et al.	[35]	2018	Italy	S/CM/F	20	10/N/10
Pignatelli P. et al.	[41]	2021	Italy	S/CM	36	36/N/N
Tran H. et al.	[39]	2022	Vietnam	S/CM	63	42/21/N
Zhang X. et al.	[38]	2022	China	S	324	207/43/41/ HYP 33
Guven D. et al.	[36]	2019	Turkey	S	148	71/N/77

S—saliva samples; F—fecal samples; CM—colonic mucosa/tumor tissue; CRC—colorectal cancer; CRA—colorectal adenoma; HC—healthy control group; HYP—hyperplastic polyp.

**Table 2:** Distribution of the participants according to stage of disease, age, and sex.

Author	Ref. Nr.	Stage of Disease	Nr./Stage (0/I/II/III/IV)	Age Median CRC/CRA/HC	Gender Male % CRC/CRA/HC	Gender—*p* Value
Zhang S. et al.	[37]	I,II,III,IV	0/24/66/60/11	59.2/51.8/50.7	67/59/53	0.192
Loftus M. et al.	[31]	III,IV	0/0/0/52/22	61/N/62	58/N/56	-
Rezasoltani S. et al.	[32]	0,I	Not mentioned	age 50 or above	not specified	-
Flemer B. et al.	[33]	I,II,III,IV	Cannot be calculated on SF	66/65.3/56.2	61/83/66.4	-
Wang Y. et al.	[42]	Not mentioned	Not mentioned	63.9/N/52.17	17.56/N/15.5	0.605
Yang Y. et al.	[43]	Not mentioned	Not mentioned	50–59 (42%)/N/50–59 (40%)	40.26/N/40.13	1.0
Idrissi J. et al.	[44]	Any	Not mentioned	63.9/N/60.4	82/N/48	-
Nosho K. et al.	[40]	I,II,III,IV	0/56/160/235/60	67.1/N/N	56/N/N	0.075
Uchino Y. et al.	[34]	I,II,III,IV	0/26 (I,II)/26(III,IV)	88.52/N/54.49	63.5/N/51	0.2
Russo E. et al.	[35]	I,II,III,IV	0/3/2/4/1	age 71–95/N/63-86	40/N/60	-
Pignatelli P. et al.	[41]	Any	1/10/10/11/3/ unknown 1	67.17/N/N	47.22/N/N	-
Tran H. et al.	[39]	II,III,IV	0/0/18/20/4	64/60/N	62/76/N	0.39
Zhang X. et al.	[38]	I,II,III,IV	78 (I,II)/129 (III,IV)	age < 63 (102 CASES), >63 (105 CASES)/N/N	53.6%/N/N	0.56
Guven D. et al.	[36]	I,II,III,IV	0/4/16/24/27	59/N/56	64.8/N/48.1	-

CRC—colorectal cancer; CRA—colorectal adenoma; HC—healthy control group.

**Table 3:** Biomarker identification and detection.

Author	Analytical Method	HC Group	Comparation	Biomarker	*p* Value	AUC (95% CI)
Zhang S. et al. [37]	DNA EXTRACION+16S rRNA SEQUENCE	YES	CRC vs. CRA VS HC	5 OTU, 3 phyla, 2 genera	under 0.05	0.8374
Loftus M. et al. [31]	WGS	YES	CRC vs. HC	8 HPS, 10 SES	under 0.05	0.87
Rezasoltani S. et al. [32]	DNA EXTRACION+16S rRNA SEQUENCE	YES	CRC vs. HC	1 genera(saliva) 3 genera(stool)	S under 0.05; F over 0.05	not determined
Flemer B. et al. [33]	DNA EXTRACION+16S rRNA SEQUENCE	YES	CRC vs. CRA VS HC	17 OTU	over 0.05 (0.08) S&F	0.94
Wang Y. et al. [42]	DNA EXTRACION+16S rRNA SEQUENCE	YES	CRC vs. HC	9 taxa (5 oral type II, 4 enterotype III)+ 4OTUS of Fusobacterium (3CM,1S)	over 0.05	not determined
Yang Y. et al. [43]	DNA EXTRACION+16S rRNA SEQUENCE	YES	CRC vs. HC	2 paodontal pathogens, 11 common taxa, 16 rare taxa	under 0.05	not determined
Idrissi J. et al. [44]	qPCR	YES	CRC vs. HC	F. nucleatum	over 0.05	not determined
Nosho K. et al. [40]	qPCR	NO	no comparation	F. nucleatum	-	not determined
Uchino Y. et al. [34]	DNA EXTRACION+16S rRNA SEQUENCE	YES	I/II vs. III/IV/ CRC VS HC	4 oral bacteria species	S under 0.05; F over 0.05	not determined
Russo E. et al. [35]	qPCR, NGS 16S RNA SEQUENCE	YES	CRC vs. HC	4 phyla	F, CM under 0.05, S over 0.05	not determined
Pignatelli P. et al. [41]	qPCR	NO	no comparation	F. nucleatum	CM under 0.05, S over 0.05	not determined
Tran H. et al. [39]	WGS, anaerobic culture, 16S rRNA profiling	NO	CRC vs. CRA	3 taxa	under 0.05	not determined
Zhang X. et al. [38]	multiplex qPCR	YES	CRC vs. HC,CRA,HYP	F. nucleatum	under 0.05	0.84
Guven D. et al. [36]	qPCR	YES	CRC vs. HC	F. nucleatum, Streptococcus gallolyticus	under 0.05	0.84

NGS—Next-Generation Sequencing; WGS- whole genome sequencing; qPCR—quantitative polymerase chain reaction; OTU—Operational Taxonomic Units; HPS—highly prevalent species; SES- significant elevated species; AUC- area under curve; CI—confidence interval.

## Data Availability

Data are contained within the article or Supplementary Material.

## Supplementary Materials

The following supporting information can be downloaded at: https://www.mdpi.com/article/10.3390/microorganisms11061586/s1, Figure S1: Risk of Bias and Applicability; Supplementary File S1: Search Strategy; Supplementary File S2: Bacterial species.

## References

[1] World Cancer Research Fund International (2022). Colorectal Cancer Statistics. https://www.wcrf.org/cancer-trends/colorectal-cancer-statistics/.

[2] O’Connell J.B., Maggard M.A., Ko C.Y. (2004). Colon cancer survival rates with the new American Joint Committee on Cancer sixth edition staging. J. Natl. Cancer Inst..

[3] Niedermaier T., Tikk K., Gies A., Bieck S., Brenner H. (2020). Sensitivity of Fecal Immunochemical Test for Colorectal Cancer Detection Differs according to Stage and Location. Clin. Gastroenterol. Hepatol..

[4] Tinmouth J., Lansdorp-Vogelaar I., Allison J.E. (2015). Faecal immunochemical tests versus guaiac faecal occult blood tests: What clinicians and colorectal cancer screening programme organisers need to know. Gut.

[5] Imperiale T.F., Ransohoff D.F., Itzkowitz S.H., Levin T.R., Lavin P., Lidgard G.P., Ahlquist D.A., Berger B.M. (2014). Multitarget stool DNA testing for colorectal-cancer screening. N. Engl. J. Med..

[6] Zwezerijnen-Jiwa F.H., Sivov H., Paizs P., Zafeiropoulou K., Kinross J. (2023). A systematic review of microbiome-derived biomarkers for early colorectal cancer detection. Neoplasia.

[7] Sender R., Fuchs S., Milo R. (2016). Revised estimates for the number of human and Bacteria cells in the body. PLoS Biol..

[8] Kostic A.D., Gevers D., Pedamallu C.S., Michaud M., Duke F., Earl A.M., Ojesina A.I., Jung J., Bass A.J., Tabernero J. (2012). Genomic analysis identifies association of Fusobacterium with colorectal carcinoma. Genome Res..

[9] Dzutsev A., Goldszmid R.S., Viaud S., Zitvogel L., Trinchieri G. (2015). The role of the microbiota in inflammation, carcinogenesis, and cancer therapy. Eur. J. Immunol..

[10] Yu T., Guo F., Yu Y., Sun T., Ma D., Han J., Qian Y., Kryczek I., Sun D., Nagarsheth N. (2017). Fusobacterium nucleatum Promotes Chemoresistance to Colorectal Cancer by Modulating Autophagy. Cell.

[11] Tjalsma H., Boleij A., Marchesi J.R., Dutilh B.E. (2012). A bacterial driver–passenger model for colorectal cancer: Beyond the usual suspects. Nat. Rev. Microbiol..

[12] Sears C.L., Pardoll D.M. (2011). Perspective: Alpha-bugs, their microbial partners, and the link to Colon Cancer. J. Infect. Dis..

[13] Gopalakrishnan V., Spencer C.N., Nezi L., Reuben A., Andrews M.C., Karpinets T.V., Prieto P.A., Vicente D., Hoffman K., Wei S.C. (2018). Gut microbiome modulates response to anti–PD-1 immunotherapy in melanoma patients. Science.

[14] Matson V., Fessler J., Bao R., Chongsuwat T., Zha Y., Alegre M.-L., Luke J.J., Gajewski T.F. (2018). The commensal microbiome is associated with anti-PD-1 efficacy in metastatic melanoma patients. Science.

[15] Pfaffe T., Cooper-White J., Beyerlein P., Kostner K., Punyadeera C. (2011). Diagnostic potential of saliva: Current state and future applications. Clin. Chem..

[16] Segata N., Haake S.K., Mannon P., Lemon K.P., Waldron L., Gevers D., Huttenhower C., Izard J. (2012). Composition of the adult digestive tract bacterial microbiome based on seven mouth surfaces, tonsils, throat and stool samples. Genome Biol..

[17] Poutahidis T., Erdman S.E. (2016). Commensal bacteria modulate the tumor microenvironment. Cancer Lett..

[18] Yang Y., Weng W., Peng J., Hong L., Yang L., Toiyama Y., Gao R., Liu M., Yin M., Pan C. (2017). Fusobacterium nucleatum increases proliferation of colorectal cancer cells and tumor development in mice by activating toll-like receptor 4 signaling to nuclear factor-κb, and up-regulating expression of microRNA-21. Gastroenterology.

[19] Kim M., Friesen L., Park J., Kim H.M., Kim C.H. (2018). Microbial metabolites, short-chain fatty acids, restrain tissue bacterial load, chronic inflammation, and associated cancer in the colon of mice. Eur. J. Immunol..

[20] Song M., Chan A.T. (2019). Environmental factors, gut microbiota, and colorectal cancer prevention. Clin. Gastroenterol. Hepatol..

[21] Anand S., Kaur H., Mande S.S. (2016). Comparative in silico analysis of butyrate production pathways in gut commensals and pathogens. Front. Microbiol..

[22] O’Keefe S.J. (2016). Diet, microorganisms and their metabolites, and colon cancer. Nat. Rev. Gastroenterol. Hepatol..

[23] Le Bars P., Matamoros S., Montassier E., Le Vacon F., Potel G., Soueidan A., Jordana F., de la Crochetiere M.-F. (2017). The oral cavity microbiota: Between health, oral disease, and cancers of the aerodigestive tract. Can. J. Microbiol..

[24] Hayes R.B., Ahn J., Fan X., Peters B.A., Ma Y., Yang L., Agalliu I., Burk R.D., Ganly I., Purdue M.P. (2018). Association of oral microbiome with risk for incident head and neck squamous cell cancer. JAMA Oncol..

[25] Lamont R.J., Koo H., Hajishengallis G. (2018). The oral microbiota: Dynamic communities and host interactions. Nat. Rev. Microbiol..

[26] Kitamoto S., Nagao-Kitamoto H., Hein R., Schmidt T.M., Kamada N. (2020). The bacterial connection between the oral cavity and the gut diseases. J. Dent. Res..

[27] Ito M., Kanno S., Nosho K., Sukawa Y., Mitsuhashi K., Kurihara H., Igarashi H., Takahashi T., Tachibana M., Takahashi H. (2015). Association of Fusobacterium nucleatum with clinical and molecular features in colorectal serrated pathway. Int. J. Cancer.

[28] Page M.J., McKenzie J.E., Bossuyt P.M., Boutron I., Hoffmann T.C., Mulrow C.D., Shamseer L., Tetzlaff J.M., Akl E.A., Brennan S.E. (2021). The PRISMA 2020 statement: An updated guideline for reporting systematic reviews. BMJ.

[29] Whiting P.F., Rutjes A.W., Westwood M.E., Mallett S., Deeks J.J., Reitsma J.B., Leeflang M.M.G., Sterne J.A.C., Bossuyt P.M.M., the QUADAS-2 Group (2011). QUADAS-2: A revised tool for the quality assessment of diagnostic accuracy studies. Ann. Intern. Med..

[30] Review Manager (RevMan) (2020). Computer Program, Version 5.4.

[31] Loftus M., Hassouneh S.A., Yooseph S. (2021). Bacterial community structure alterations within the colorectal cancer gut microbiome. BMC Microbiol..

[32] Rezasoltani S., Aghdaei H.A., Jasemi S., Gazouli M., Dovrolis N., Sadeghi A., Schlüter H., Zali M.R., Sechi L.A., Feizabadi M.M. (2022). Oral Microbiota as Novel Biomarkers for Colorectal Cancer Screening. Cancers.

[33] Flemer B., Warren R.D., Barrett M.P., Cisek K., Das A., Jeffery I.B., Hurley E., O’Riordain M., Shanahan F., O’Toole P.W. (2018). The oral microbiota in colorectal cancer is distinctive and predictive. Gut.

[34] Uchino Y., Goto Y., Konishi Y., Tanabe K., Toda H., Wada M., Kita Y., Beppu M., Mori S., Hijioka H. (2021). Colorectal Cancer Patients Have Four Specific Bacterial Species in Oral and Gut Microbiota in Common-A Metagenomic Comparison with Healthy Subjects. Cancers.

[35] Russo E., Bacci G., Chiellini C., Fagorzi C., Niccolai E., Taddei A., Ricci F., Ringressi M.N., Borrelli R., Melli F. (2018). Preliminary Comparison of Oral and Intestinal Human Microbiota in Patients with Colorectal Cancer: A Pilot Study. Front. Microbiol..

[36] Guven D.C., Dizdar O., Alp A., Akdoğan Kittana F.N., Karakoc D., Hamaloglu E., Lacin S., Karakas Y., Kilickap S., Hayran M. (2019). Analysis of Fusobacterium nucleatum and Streptococcus gallolyticus in saliva of colorectal cancer patients. Biomark. Med..

[37] Zhang S., Kong C., Yang Y., Cai S., Li X., Cai G., Ma Y. (2020). Human oral microbiome dysbiosis as a novel non-invasive biomarker in detection of colorectal cancer. Theranostics.

[38] Zhang X.T., Zhang Y., Gui X., Zhang Y., Zhang Z., Zhang X., Chen W., Zhang X., Wang Y., Zhang M. (2022). Salivary Fusobacterium Nucleatum Serves as a Potential Biomarker for the Diagnosis and Prognosis of Colorectal Cancer. Iscience.

[39] Tran H.N.H., Thu T.N.H., Nguyen P.H., Vo C.N., Doan K.V., Ngoc Minh C.N., Nguyen N.T., Duc Ta V.N., Vu K.A., Hua T.D. (2022). Tumour microbiomes and *Fusobacterium genomics* in Vietnamese colorectal cancer patients. NPJ Biofilms Microbiomes.

[40] Nosho K., Sukawa Y., Adachi Y., Ito M., Mitsuhashi K., Kurihara H., Kanno S., Yamamoto I., Ishigami K., Igarashi H. (2016). Association of Fusobacterium nucleatum with immunity and molecular alterations in colorectal cancer. World J. Gastroenterol..

[41] Pignatelli P., Iezzi L., Pennese M., Raimondi P., Cichella A., Bondi D., Grande R., Cotellese R., Di Bartolomeo N., Innocenti P. (2021). The Potential of Colonic Tumor Tissue Fusobacterium nucleatum to Predict Staging and Its Interplay with Oral Abundance in Colon Cancer Patients. Cancers.

[42] Wang Y., Zhang Y., Qian Y., Xie Y.H., Jiang S.S., Kang Z.R., Chen Y.X., Chen Z.F., Fang J.Y. (2021). Alterations in the oral and gut microbiome of colorectal cancer patients and association with host clinical factors. Int. J. Cancer.

[43] Yang Y., Cai Q., Shu X.O., Steinwandel M.D., Blot W.J., Zheng W., Long J. (2019). Prospective study of oral microbiome and colorectal cancer risk in low-income and African American populations. Int. J. Cancer.

[44] Idrissi Janati A., Karp I., Von Renteln D., Bouin M., Liu Y., Tran S.D., Emami E. (2022). Investigation of Fusobacterium Nucleatum in saliva and colorectal mucosa: A pilot study. Sci. Rep..

[45] Pallag A., Rosca E., Tit D.M., Mutiu G., Bungau S.G., Pop O.L. (2015). Monitoring the effects of treatment in colon cancer cells using immunohistochemical and histoenzymatic techniques. Rom. J. Morphol. Embriol..

[46] Ghitea T.C., Vlad S., Birle D., Tit D.M., Lazar L., Nistor-Cseppento C., Behl T., Bungau S. (2020). The influence of diet therapeutic intervention on the sarcopenic index of patients with metabolic syndrome. Acta Endocrinol..

[47] Negrut N., Khan S.A., Bungau S., Zaha C., Corb Aron R.A., Bratu O., Diaconu C.C., Ionita-Radu F. (2020). Diagnostic challenges in gastrointestinal infections. Rom. J. Mil. Med..

[48] Zaha D.C., Bungau S., Uivarosan D., Tit D.M., Maghiar T.A., Maghiar O., Pantis C., Fratila O., Rus M., Vesa C.M. (2020). Antibiotic consumption and microbiological epidemiology in surgery departments: Results from a single study center. Antibiotics.

[49] Zaha D.C., Bungau S., Aleya S., Tit D.M., Vesa C.M., Popa A.R., Pantis C., Maghiar O.A., Bratu O.G., Furau C. (2019). What antibiotics for what pathogens? The sensitivity spectrum of isolated strains in an intensive care unit. Sci. Total Environ..

[50] Koliarakis I., Messaritakis I., Nikolouzakis T.K., Hamilos G., Souglakos J., Tsiaoussis J. (2019). Oral bacteria and intestinal dysbiosis in colorectal cancer. Int. J. Mol. Sci..

