# Supplementary material for: Exploring the Potential of Oral Microbiome Biomarkers for Colorectal Cancer Diagnosis and Prognosis: A Systematic Review
Source: Microorganisms. 2023 Jun 15;11(6):1586. doi: 10.3390/microorganisms11061586 (PMC10305386; doi:10.3390/microorganisms11061586)
Supplement: Supplementary file 1 [file microorganisms-11-01586-s001.zip › Supplementary file S1.pdf]

Filter ARTICLE

WOS
((TI=(colorectal cancer) OR TI=(colonic cancer)) AND (TS=(Periodontal pathogens) OR TS=(oral microbiota) OR TS=(oral microbiome))) – 69 results

By expanding the search:

((TI=(colorectal cancer) OR TI=(colonic cancer)) AND (ALL=(periodontal pathogens) OR ALL=(oral microbiota) OR ALL=(oral microbiome))) -81 results (10 se)

Science direct
colorectal cancer or colonic cancer and oral microbiota or oral microbiome or periodontal pathogens
– 21 studies

scopus
TITLE (colorectal AND cancer) OR TITLE (colonic AND cancer) AND TITLE-ABS (periodontal AND pathogens) OR TITLE-ABS (oral AND microbiota) OR TITLE-ABS (oral AND microbiome).
– 52 studies

PubMed
((COLORECTAL CANCER[Title]) OR (COLONIC CANCER[Title])) AND ((PERIODONTAL PATHOGENS[Title/Abstract]) OR (ORAL MICROBIOME[Title/Abstract]) OR (ORAL MICROBIOTA[Title/Abstract]))
-20 studies
