# Supplementary material for: Exploring the Potential of Oral Microbiome Biomarkers for Colorectal Cancer Diagnosis and Prognosis: A Systematic Review
Source: Microorganisms. 2023 Jun 15;11(6):1586. doi: 10.3390/microorganisms11061586 (PMC10305386; doi:10.3390/microorganisms11061586)
Supplement: Supplementary file 1 [file microorganisms-11-01586-s001.zip › Supplementary file S2.pdf]

BACTERIAL SPECIES

1.
2. bacterial abundance was higher in the CRC group :

    phyla Fusobacteria and Bacteroidetes

    the genera Fusobacterium, Prevotella, and Veillonella

the diagnostic value of the CRC oral microbiome -five OTU markers

g__Cyanobacteria_unclassified
g__Veillonella
g__Selenomonas
g__Fusobacterium
g__Gemella

2.

The 10 species significantly elevated in relative abundance within CRC :

Parvimonas micra (qvalue = 3.09e-09),

Peptostrepto- coccus stomatis (qvalue = 4.51e-08),

Gemella morbillorum (qvalue = 4.55e-08),

Fusobacterium nucleatum (qvalue = 1.08e-06),

Streptococcus anginosus (qvalue = 1.13e-03),

Dialister pneumosintes (qvalue=1.37e-03),

Peptostreptococcus anaerobius (qvalue = 4.74e-03),

Streptococcus sp. KCOM 2412 (Streptococcus periodonticum) (qvalue = 7.18e-03),

Ruminococcus torques (qvalue = 1.55e-02),

Filifactor alocis (qvalue = 2.85e-02)

The eight HPS unique to the CRC associated group :

Intestinibacillus massiliensis,

Prevotella copri,

Haemophilus parainfluenzae,

Ruminococcus bicirculans,

Streptococcus mitis,

Neglecta timonensis,

Bifidobacterium catenulatum

Anaerotignum neopropionicum.

3.

top three genera that were differentially abundant in the saliva of CRC patients vs. healthy controls :

Eubacterium spp., Bifidobacterium spp., and Fusobacterium spp

fecal microbiome in CRC and the HC showed a total of six genera, three of which are associated with HC fecal and three with CRC fecal.

CRC fecal samples : genera Ruminococcus-torques-group, Granulicatella, and Ruminococcus-gauvreauii-group.

4.

The 10 most abundant bacterial genera across all oral swab

Streptococcus (30.7% of all assigned reads), Haemophilus (14.2%), Neisseria (8.8%), Prevotella (6.6%), Fusobacterium (5.4%), Veillonella (5.4%), Leptotrichia (3.9%), Rothia (3.9%), Actinomyces (2.9%) and Porphyromonas (2.4%)

17 OTUs that were shared between the oral cavity and CRC and polyp samples;

The two tumour-associated bacterial coabundance groups (CAGs) eg, F. nucleatum, Parvi- monas micra, Peptostreptococcus stomatis, Dialister pneu- mosintes and - oral pathogen CAG and comprising seven OTUs in total. The second CAG : Actinomyces, Haemophilus, Rothia, Streptococcus and Veilonella spp.,[34] genera also associated with relatively healthy tooth pockets[32] (so-called biofilm CAG; 10 OTUs)

5.

the saliva of CRC patients : The relative abundance of Bacteroides, Streptococcus and Desulfovibrio genera was increased; level of Porphyromonas gingivalis was significantly elevated in CRC patients (Mann-Whitney U test P value = .013)

Firmicutes and Bacteroides in the mucosa microbiome were more abundant in CRC

CRC was dominated by type II oral-type: overabundance of Streptococcus, Neisseria, Porphyromonas, Prevotella_7 and Haemophilus genus.

CRC group was mainly dominated by type III enterotype, which was represented by Fusobacterium, Bacte- roides, Streptococcus and Peptostreptococcus.

Fusobacterium genus in CRC were higher than those in HC. These OTUs mainly belonged to Fusobacterium mortiferum, Fusobacterium varium, Fusobacterium per- iodonticum and unclassified Fusobacterium. The relative abundance of OTU 2327 assigned to Fusobacterium mortiferum, was more abundant in the stool of the CRC group

6.

ORAL CRC: Treponema denticola and Prevotella intermedia $p < 0.05$

Phylum Actinobacteria

Family Bifidobacteriaceae	0.09%	0.05%	1.10 (1.01–1.19)	0.03	1.00
Phylum Bacteroidetes					
Species Prevotella denticola	0.14%	0.07%	1.11 (1.02–1.20)	0.02	1.00
Species Prevotella melaninogenica	2.03%	2.12%	0.91 (0.84–0.99)	0.04	1.00
Species Prevotella sp. oral taxon 300	0.04%	0.02%	1.13 (1.01–1.26)	0.04	1.00
Phylum Firmicutes					
Family Carnobacteriaceae	1.22%	1.39%	0.85 (0.72–0.99)	0.04	1.00
Family Streptococcaceae	33.45%	36.23%	0.73 (0.56–0.96)	0.02	1.00
Genus Streptococcus	33.40%	35.92%	0.73 (0.55–0.96)	0.02	1.00
Species S. sp. oral taxon 058	16.66%	18.59%	0.79 (0.67–0.94)	7.87×10^{-3}	0.42
Family Erysipelotrichaceae	0.09%	0.09%	0.87 (0.76–0.99)	0.04	1.00
Genus Solobacterium	0.07%	0.07%	0.87 (0.76–0.98)	0.02	1.00
Species S. moorei	0.07%	0.07%	0.87 (0.77–0.99)	0.03	1.00

Phylum Actinobacteria					
Species Bifidobacterium dentium	58.87%	50.54%	1.46 (1.04–2.07)	0.03	1.00
Phylum Bacteroidetes					
Species Prevotella sp. oral taxon 304	33.77%	26.03%	1.59 (1.09–2.33)	0.02	1.00
Phylum Firmicutes					
Genus Peptococcus	60.61%	52.06%	1.46 (1.02–2.08)	0.04	1.00
Genus Anaeroglobus	69.70%	60.74%	1.48 (1.04–2.10)	0.03	1.00
Genus Mitsuokella	44.16%	36.01%	1.52 (1.08–2.14)	0.02	1.00

Species Lactobacillus salivarius	46.75%	38.18%	1.46 (1.03–2.08)	0.03	1.00
Species Eubacterium yurii	37.66%	29.93%	1.46 (1.01–2.10)	0.04	1.00
Phylum Proteobacteria					
Family Burkholderiaceae	66.67%	55.97%	1.62 (1.14–2.30)	7.40×10^{-3}	0.68
Genus Lautropia	66.23%	54.45%	1.72 (1.20–2.45)	2.88×10^{-3}	0.26
Species Neisseria oralis	42.42%	34.71%	1.42 (1.01–2.00)	0.04	1.00
Species Campylobacter sp. oral taxon 044	51.52%	42.08%	1.58 (1.12–2.24)	0.01	0.92
Phylum Spirochaetes					
Species Treponema lecithinolyticum	44.59%	33.62%	1.76 (1.23–2.53)	2.11×10^{-3}	0.19
Species Treponema sp. oral taxon 250	25.97%	20.17%	1.66 (1.07–2.56)	0.02	1.00
Phylum SR1	54.98%	41.65%	1.76 (1.25–2.47)	1.09×10^{-3}	0.10
Species SR1_[G-1] sp. oral taxon 345	33.77%	26.90%	1.50 (1.01–2.23)	0.04	1.00
Species SR1_[G-1] sp. oral taxon 874	41.13%	29.07%	1.75 (1.24–2.48)	1.58×10^{-3}	0.14

7. Idrissi Janati A, Karp I, Von Renteln D, Bouin M, Liu Y, Tran SD, Emami E

F. nucleatum in saliva from both case and control groups,

F. nucleatum in colorectal mucosa LOW in the controls, and it was even lower in the cases.

8. F. nucleatum positivity was detected in 44 (8.6%) of the 511 Japanese patients with colorectal cancer

9. Peptostreptococcus stomatis, Streptococcus anginosus, Solobacterium moorei, and Streptococcus koreensis,- saliva and stool of patients with CRC

10.

three main bacterial phyla : Firmicutes (39.18%), Bacteroidetes (30.36%), and Proteobacteria (10.65%)

CT - Proteobacteria, Bacteroidetes, and Firmicutes).

saliva : Actinobacteria, Saccharibacteria, Proteobacteria (Beta class), Fusobacteria, Firmicutes (mainly Negativicutes and Bacilli), and Bacteroidetes (exclusively represented by members of Flavobacteriia class and Prevotellaceae family)

stool : Bacteroidetes (in particular the Bacteroidia class) and Firmicutes (mainly Clostridia and Erysipelotrichia classes)

biopsy Proteobacteria, mainly Delta, Alpha, and Gamma classes, Planctomycetes, and Firmicutes (namely Lachnospiraceae family and Clostridiales family XI), enriched in members of Fusobacterium genus

11.

of Fn was detected in both the oral cavity and matched cancer tissue and adjacent non-neoplastic mucosa. Pg present in the oral cavity

12.

bacteria of putative oral origin overabundant in the tumour microbiomes:

Gemella

Peptostreptococcus,

F. nucleatum,

Leptotrichia,

Selenomonas sputigena

Campylobacter rectus

WGS- F. nucleatum high in advanced CRC stages p-value < 0.05

13.

Fn DNA was increased in the saliva of the CRC

14.

Saliva - Fn ($p = 0.001$) and Sg ($p < 0.001$) compared with controls.

Fn : 69 of 71 cases (97.2%) in the patient group

74 of 77 subjects (96.1%) in the control group ($p > 0.99$)

Mean Fn amount was higher in patient saliva samples compared with controls $p = 0.001$

Sg :22 of 71 patients (31%)

21 of 77 controls (27.3%)

The amount of Sg was found to have diagnostic value $p < 0.001$

Pg was detected in 54 patients (76.1%) and 58 controls (75.3%).
