# Supplementary figures and images for: Exploring the Potential of Oral Microbiome Biomarkers for Colorectal Cancer Diagnosis and Prognosis: A Systematic Review
Source: Microorganisms. 2023 Jun 15;11(6):1586. doi: 10.3390/microorganisms11061586 (PMC10305386; doi:10.3390/microorganisms11061586)

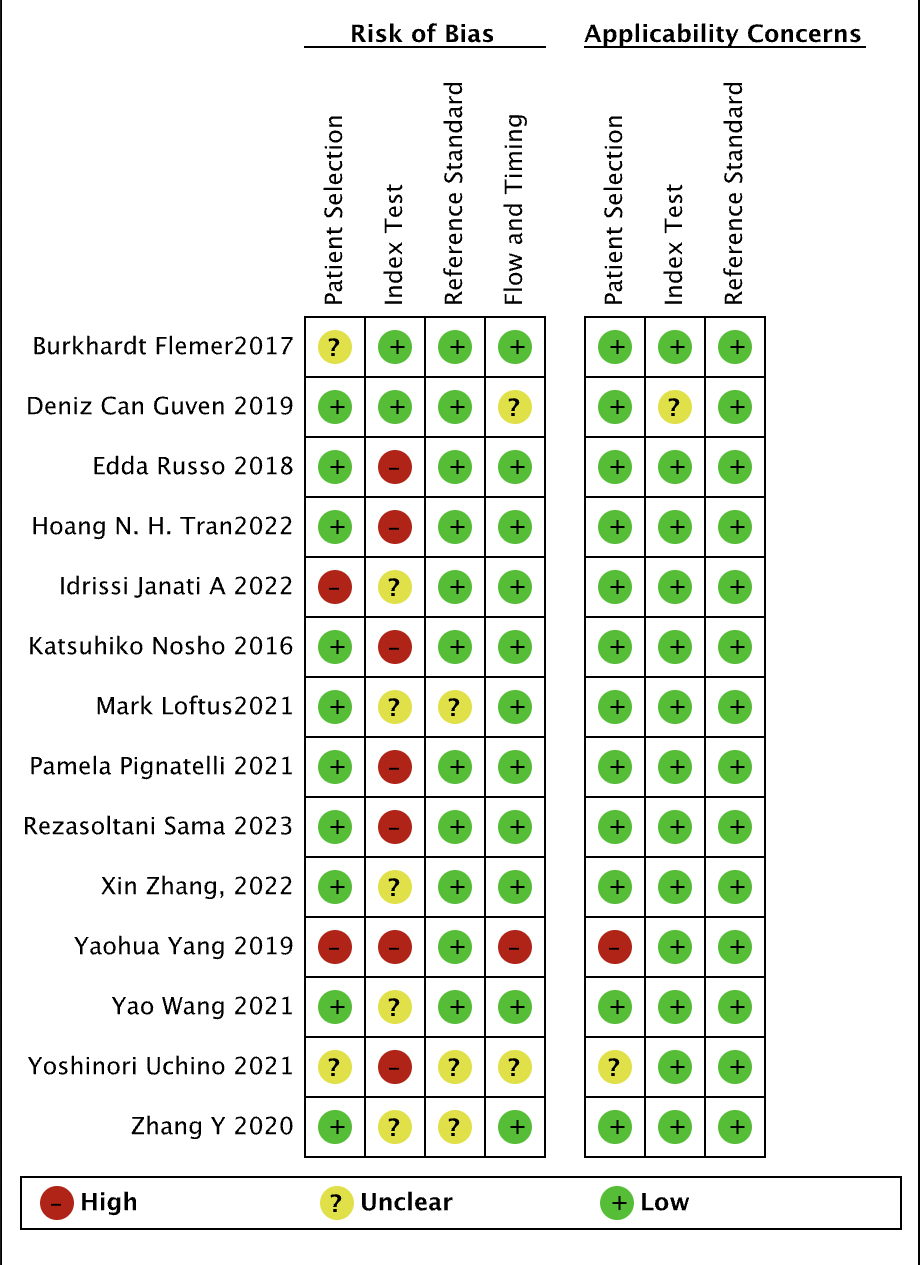

Supplement: Supplementary file 1 [file microorganisms-11-01586-s001.zip › Supplementary Figure 1.png]
